# Associations Between Combinations of 24-Hour Movement Behaviors and Dietary Outcomes in Children and Adolescents: A Systematic Review

**DOI:** 10.3390/nu16213678

**Published:** 2024-10-29

**Authors:** Nan Zeng, Shan Jiang, Abigail Ringer, Catalina Pacheco, Chunmei Zheng, Sunyue Ye

**Affiliations:** 1 Prevention Research Center, Department of Pediatrics, School of Medicine, University of New Mexico Health Sciences Center, Albuquerque, NM 87131, USA; 2 Department of Exercise and Health Sciences, Manning College of Nursing and Health Sciences, University of Massachusetts Boston, Boston, MA 02125, USA; 3 Department of Sports Science and Physical Education, The Chinese University of Hong Kong, Hong Kong; sjiang99@link.cuhk.edu.hk; 4 College of Population Health, University of New Mexico Health Sciences Center, Albuquerque, NM 87131, USA; 5 Department of Psychology, University of New Mexico, Albuquerque, NM 87131, USA; 6 School of Physical Education, Shandong University, Jinan 250061, China; 7 Institute of Child Development, Jiaxing University, Jiaxing 314001, China

**Keywords:** adolescents, breakfast, children, fruits, Mediterranean diet, physical activity, sedentary time, sleep, vegetables

## Abstract

**Background/Objectives**: Twenty-four-hour movement behaviors, including physical activity, sedentary time, and sleep, are associated with a range of health outcomes. However, the relationship between the combinations of these behaviors and dietary outcomes in young populations remains unclear. We conducted a systematic review to explore how combined movement behaviors are linked to dietary outcomes in children and adolescents. **Methods**: We searched MEDLINE, Embase, and PsycINFO from January 2017 to July 2024, including studies that were (1) written in English, (2) published in peer-reviewed journals, (3) quantitatively analyzed two or three movement behaviors with dietary outcomes, and (4) focused on populations aged between 0 and 18 years. **Results**: Ten cross-sectional studies met these criteria. The majority of studies (6 out of 10) assessed all three movement behaviors, although sleep was less frequently examined, appearing in only 4 of the studies. Our findings indicated that adhering to all movement recommendations was consistently associated with healthier dietary patterns, including a higher consumption of fruits, vegetables, nuts, fish, and cereals, and a lower intake of sweets and pastries. Additionally, adhering to physical activity and sedentary time guidelines was linked to improved dietary habits in children, including more fruit and vegetable intake and less sugary drink and snack consumption. Conversely, higher sedentary time and inadequate sleep were linked to poorer dietary outcomes, particularly lower fruit intake and hydration. **Conclusions**: This review suggests that meeting movement behavior guidelines correlates with healthier dietary outcomes in children and adolescents. However, given the limited number of studies and their cross-sectional design, further high-quality research, including longitudinal and intervention studies, is needed to clarify causal relationships.

## 1. Introduction

Physical activity, sedentary time, and sleep are time-dependent behaviors that initiate distinct physiological processes and interact throughout a 24-hour period to create a cumulative biological impact [1]. These behaviors are known as “movement behaviors”. Developed by the Canadian Society for Exercise Physiology (CSEP) in partnership with other health organizations in 2016, Canada became the first country to release the *24-Hour Movement Guidelines for Children and Youth (ages 5–17)* [2]. These guidelines emphasize the integration of all movement behaviors over a 24-hour period, rather than addressing them in isolation, and promote a balanced approach to activity [3]. In 2017, the *24-Hour Movement Guidelines for the Early Years (0–4 years)* were also released, specifically tailored to meet the developmental needs of younger children [4]. The guidelines encourage children and youth to move more, sit less, and sleep well for optimal health and development. Since their release, these guidelines have become a key framework for researchers studying childhood development. They establish standardized benchmarks for assessing the connections between physical activity, sedentary time, and sleep, helping pediatric researchers better understand how these movement behaviors influence long-term health outcomes [5]. Furthermore, the guidelines are a valuable resource for parents, caregivers, health professionals, and policymakers, playing a key role in promoting healthy development in children and adolescents [6].

Previous review evidence has highlighted the associations between movement behaviors and various health outcomes in children and adolescents. The consensus is that (1) higher levels of physical activity are positively linked to improvements in physical, psychological, social, and cognitive health indicators, as well as healthier eating behaviors [7,8,9,10,11]; (2) total sedentary time is linked to health indicators such as adiposity, cardiometabolic health, motor and cognitive development, fitness, and dietary intake [12,13,14,15]; and (3) sleep duration is related to outcomes like obesity, dietary habits, cognition, cardiometabolic metrics, and psychosocial health [16,17,18]. However, these movement behaviors have largely been studied individually in relation to health outcomes rather than examining their combined effects. This fragmented approach overlooks the potential interactions between physical activity, sedentary time, and sleep, which may collectively influence overall health than when considered independently [3]. Understanding the combined impact of these behaviors is essential, as it reflects the real-world dynamics of how children and adolescents engage in movement throughout a 24-hour period [2]. This gap in research, therefore, highlights the need for more comprehensive studies that assess the integrated effects of these behaviors on health outcomes.

Several review studies have examined the relationships between combined movement behaviors and various health indicators in children and adolescents. Kuzik et al. (2017), for example, found that the most favorable combinations of low sedentary time and high physical activity were associated with enhanced motor development, improved physical fitness, and reduced adiposity in preschool children [19]. Sampasa–Kanyinga et al. (2020) observed that meeting all three 24-hour movement behavior recommendations was positively associated with improved mental health indicators in children and adolescents [20]. Wilhite (2023) concluded that improved physical health, psychological well-being, and educational outcomes were associated with higher levels of physical activity and lower levels of sedentary time [21]. Building upon these promising findings, further review synthesizing how the integration of movement behaviors influences other health outcomes in young populations is warranted.

In recent years, the link between meeting 24-hour movement guidelines and dietary outcomes has attracted growing research interest, particularly due to the interplay between physical activity, sedentary time, sleep, and overall health. Dietary intake is closely linked to movement behaviors, with evidence showing that poor diet and unhealthy movement patterns can lead to obesity, cardiometabolic diseases, and other chronic conditions later in life [13]. In addition, the integration of movement behaviors with dietary habits is essential for promoting balanced energy intake and expenditure, which supports proper growth, development, and long-term health in children and adolescents [22]. Despite growing evidence, it remains largely unclear whether meeting specific or all combinations of movement behavior recommendations is more beneficial for children’s dietary patterns than meeting individual behaviors alone. Understanding this relationship is key to developing comprehensive health management strategies for children and adolescents, as promoting balanced movement behaviors and healthy eating is crucial for preventing chronic health conditions and supporting long-term well-being. Therefore, the purpose of this systematic review is to synthesize existing evidence on the relationship between combinations of 24-hour movement behaviors and dietary outcomes in children and adolescents. This review aims to fill a gap in the literature by exploring how these behaviors together influence dietary behaviors, with the goal of informing future interventions and public health guidelines that support optimal growth and development in young populations.

## 2. Materials and Methods

### 2.1. Study Protocol and Registration

This review was carried out in alignment with the Preferred Reporting Items for Systematic Reviews and Metanalysis (PRISMA) 2020 guidelines for reporting systematic reviews and metanalyses [23]. This review was registered with the International Prospective Register of Systematic Reviews (PROSPERO) (http://www.crd.york.ac.uk/prospero/, accessed on 7 May 2024; Registration no. CRD42024582115).

### 2.2. Eligibility Criteria

Only quantitative articles published in peer-reviewed journals and written in English were included. The Population, Intervention, Comparison, Outcomes, and Study Design (PICOS) framework [24] was applied to identify key concepts in the research question beforehand, helping to guide and streamline the search process.

#### 2.2.1. Participants/Population

Only studies involving healthy children and adolescents from 0 to 18 years were eligible for inclusion. This includes infants (0–1 year), toddlers (1–2 years), preschoolers (3–5 years), children (6–12 years), and adolescents (13–18 years). Eligible studies required a mean participant age between one month and 17 years, 11 months, regardless of the full age range. For example, a study with participants aged from 7 to 20 years but a mean age of 11 years was included. Studies were excluded if they focused on clinical samples (e.g., children with disabilities) or if physical activity, sedentary time, or sleep were not assessed at least once within the 0 to 18-year age range.

#### 2.2.2. Interventions/Exposures

Studies were eligible if they reported on 24-hour movement behaviors: physical activity, sedentary time, and sleep duration. Both objective measures (e.g., accelerometry and pedometers) and subjective measures (proxy- or self-report) were considered. For experimental studies, interventions had to focus on two or three movement behaviors, but an observed change in these behaviors was not necessary for inclusion. For observational studies, the exposure should involve any combination of two or three movement behaviors. Relevant interventions/exposures were defined by the duration, type, frequency, and intensity associated with these behaviors.

#### 2.2.3. Comparators/Control

The comparator involved different durations and combinations of the three-movement behaviors, with no requirement for a comparator or control group for inclusion.

#### 2.2.4. Outcomes

Dietary behaviors represented our primary outcome of interest, including food consumption, fruits, vegetables, nutrition, eating habits, food intake, energy intake, dietary patterns, beverage (or water) intake, healthy eating index, portion size, and appetite.

#### 2.2.5. Study Design

There were no restrictions on study designs or statistical analysis methods. However, case studies and grey literature (e.g., reports, government documents, unpublished manuscripts) were excluded because they often lack peer review and rigorous methodological evaluation, which can compromise the reliability and validity of their findings [25].

### 2.3. Information Sources and Search Strategy

The corresponding author (N.Z.) developed the search strategies that were created in consultation with a reference librarian and search specialist and were based on previously developed and validated searches exploring the relationships between combinations of 24-hour movement behaviors (i.e., physical activity, sedentary time, and sleep) and health outcomes in children and adolescents [7,19,20,26]. Literature was gathered through searches in MEDLINE, Embase, and PsycINFO using the Ovid interface. Given that the first version of the 24-Hour Movement Guidelines was released in 2016, the search period was set from January 2017 to July 2024, and no eligible studies were conducted before 2017. The search process was assisted by two coauthors (A.R. and C.P.) (see Supplementary Materials for a full description of the strategy used for each database).

### 2.4. Data Extraction

To ensure high interrater reliability, a pilot literature selection was conducted. The lead author (N.Z.) initially retrieved all relevant articles based on the established inclusion and exclusion criteria. The extracted data were organized into a secure Excel sheet, which was then shared with the other authors. A comprehensive list of articles meeting the criteria was compiled in a Microsoft Excel (Version 2021) spreadsheet (Microsoft Corporation, Redmond, WA, USA) and distributed among the research team. The screening process was conducted in two rounds, with three authors (S.J., C.Z., and S.Y.) participating independently. In the first round, the titles, abstracts, and keywords of all retrieved studies were reviewed to determine their suitability, following the removal of duplicates. The second round involved a full-text review of articles that were either potentially eligible or had unclear eligibility based on the initial screening. Each study was thoroughly assessed against the predefined criteria, with any disagreements in the screening process resolved through discussion until agreement was achieved. The entire selection process was carefully documented to facilitate the creation of a PRISMA flow diagram. Data extraction was conducted by two independent reviewers (N.Z. and S.J.) using standardized data extraction from Table 1. The reviewers compared their extracted data and resolved any discrepancies through discussion with a third reviewer until reaching a consensus. It is important to note that we only reported significant findings on the combinations of movement behaviors and dietary outcomes, and non-reported relationships may be due to the authors either not examining those relationships or finding them to be non-significant.

### 2.5. Quality Assessment and Risk of Bias

The Grading of Recommendations Assessment, Development, and Evaluation (GRADE) [36] was used to evaluate the collective quality of evidence by study design across studies. This framework classifies the quality of evidence into four distinct levels: “high”, “moderate”, “low”, and “very low”. Randomized studies are initially assigned a high rating, whereas all other experimental and observational studies start with a low rating. Evidence quality may be downgraded based on five criteria: risk of bias, imprecision, inconsistency, indirectness, and publication bias [36]. On the other hand, evidence quality can be upgraded when a large effect size is present, a dose–response relationship is observed, or if all plausible confounders would reduce the treatment effect [36].

The risk of bias for each outcome was assessed according to the guidelines in the Cochrane Handbook [37]. For observational studies, we focused on screening for potential sources of bias in the overall study, including selection bias (inappropriate sampling), performance bias (flawed measurement of exposure), attrition bias (incomplete follow-up or high loss to follow-up), and selective reporting bias (selective or incomplete outcome reporting). Detection bias (flawed measurement of outcomes) was assessed for each main outcome, along with other potential sources of bias [7,9,36].

Both exposures (i.e., physical activity, sedentary time, sleep) and dietary outcomes (e.g., food consumption, energy intake) were evaluated for measurement-related biases. When performance bias was solely attributed to selection bias due to convenience sampling, it was not considered a “serious risk of bias”. All assessments were conducted by two reviewers (N.Z and S.J.) and verified by the broader review team, with any disagreements resolved through discussion.

### 2.6. Data Synthesis

Metanalyses were considered if the data showed sufficient homogeneity in methodological, statistical, and clinical characteristics. While it is possible to conduct a metanalysis for various movement and dietary outcomes using different assessment methods, this approach can introduce significant heterogeneity and potential biases, potentially undermining the validity and reliability of the metanalytic conclusions. Therefore, a metanalysis was not feasible for this review. As a result, a narrative synthesis was performed, with all included studies given equal weight and organized by study design, health outcomes, and the combination of movement behaviors (e.g., physical activity and sedentary time).

## 3. Results

**Figure 1:**
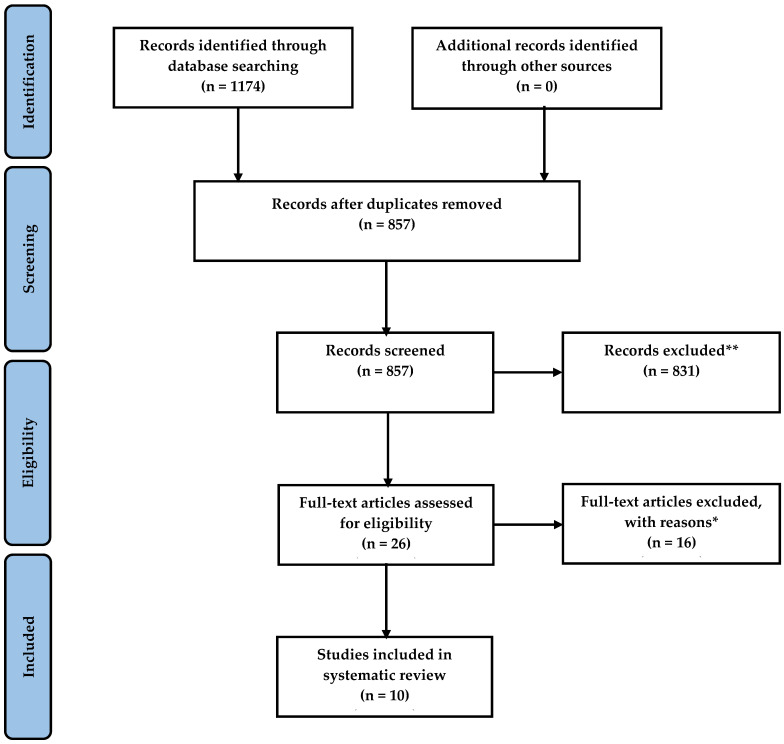
PRISMA flowchart of included studies. ** Records were excluded for the following reasons: irrelevant topics or not related to dietary outcomes, not reporting on movement behaviors, not focused on populations aged between 0 and 18 years. Some articles were excluded for more than one of these reasons. * Articles were excluded for the following reasons: not reporting a combination of movement behaviors (n = 10), not reporting the relationship between movement behaviors and dietary outcomes (n = 5), ineligible age group (n = 1).

A PRISMA flowchart illustrating the article selection process is shown in Figure 1. The electronic database search initially identified 1174 records: 627 from Medline, 462 from EMBASE, and 85 from PsycINFO. After removing duplicates, 857 unique records were subjected to title and abstract screening. Following this process, 831 records were excluded because of the following reasons: irrelevant topics, non-reporting of dietary outcomes, lack of focus on movement behaviors, and ineligible age group. In the next phase, 26 full-text articles were assessed for eligibility. Sixteen studies were excluded for not reporting on combinations of movement behaviors (n = 10), not examining the relationship between movement behaviors and dietary outcomes (n = 5), and not including participants aged between 0 and 18 years (n = 1). Ultimately, ten studies met the inclusion criteria and were included in this systematic review.

Table 1 presents the characteristics of all ten included studies. Collectively, all included studies were cross-sectional in design; these studies were from five countries: Brazil (n = 1), Czech Republic (n = 1), Canada (n = 2), Greece (n = 1), and Spain (n = 5). This distribution indicates that researchers from Spain are leading efforts in this field, contributing to the majority of studies on the relationship between 24-hour movement behaviors and dietary outcomes. Sample sizes ranged from 242 participants in the smallest study [32] to 177,091 participants in the largest study [38]. Participant ages spanned from 0 to 20 years, with an average of 11.8 years. The youngest group, consisting of preschool children aged between 4 and 5 years, was included in one study [29]. All other studies focused on either school-aged children, adolescents, or mixed-age groups. Physical activity was measured via accelerometers in four studies [27,30,31,35], while others relied on self-reported or parent-reported questionnaires. Sedentary time was predominantly measured through screen time questionnaires, with one study employing an objective measure of sedentary time [27], while sleep duration was either self-reported or, in a few cases, measured using accelerometers [31,35]. The dietary outcomes in these ten studies were categorized into food group consumption (e.g., fruit, vegetable intake, fish, nuts, etc.) and adherence to dietary patterns. Various validated measurement tools were employed, with seven studies utilizing validated instruments [27,28,30,32,34,35,38]. Specifically, food group consumption was assessed using self-reported questionnaires [31,33], the Risk Behaviors of Adolescents questionnaire [27], the Food Frequency Questionnaire (FFQ) [35], and 24-hour dietary recalls [30]. Adherence to dietary patterns, particularly the Mediterranean diet, was evaluated using the Mediterranean Diet Quality Index [29,34,38] and the Spanish Healthy Eating Index (S-HEI) [28]. These studies utilized a mix of subjective self-reports and objective dietary records to assess various dietary outcomes across different age groups. Studies analyzing the relationship between 24-hour movement behaviors and dietary outcomes used various statistical methods. Specifically, latent class analysis explored behavioral patterns and fruit intake [27]. Logistic regression was commonly used [32,34], while generalized linear models were applied to assess assessed adherence to movement guidelines [29,30]. Multivariable logistic regression [31,33] and binary logistic regression [34,38] explored diet and behavior combinations. Finally, multilevel linear mixed models assessed overall dietary patterns [35].

The findings consistently demonstrated that meeting multiple movement behavior guidelines is strongly associated with healthier dietary patterns. On the other hand, failing to meet these guidelines, particularly for combinations involving sedentary behavior and insufficient sleep, is linked to poorer dietary outcomes. No studies reported adverse dietary outcomes for participants meeting this combination of guidelines. Overall, the studies highlight the importance of adhering to multiple movement behavior guidelines for promoting healthier dietary habits in children and adolescents.

Table 2 presents the quality assessment and absolute effects of movement behaviors on dietary behaviors for all included studies. These included ten cross-sectional studies involving 204,386 children and adolescents. According to the GRADE protocol, the quality of evidence for these studies was initially rated as low due to the observational design. Although no serious inconsistency, indirectness, or imprecision was identified across these studies, a serious risk of bias was observed for multiple reasons. Specifically, (1) the validity and reliability of outcome measures remain unknown. Physical activity was assessed subjectively through self-reported measures, with no psychometric properties reported [28,29,32,33,34,38]. One study [31] used a single item to ask students how many times per week they typically consumed (a) fruits, (b) vegetables, (c) unhealthy snacks, and (d) skipped breakfast. Another study [29], which focused on preschool-aged children, measured all movement and dietary behaviors through parent-reported data; (2) potential confounders were not adequately controlled [27,32]; (3) missing data and exclusions, along with the reasons, were not reported [16,19,27,28,29,30,31,33,34,35]. As such, the quality of evidence was downgraded from “low” to “very low” due to the serious risk of bias.

**Figure 2:**
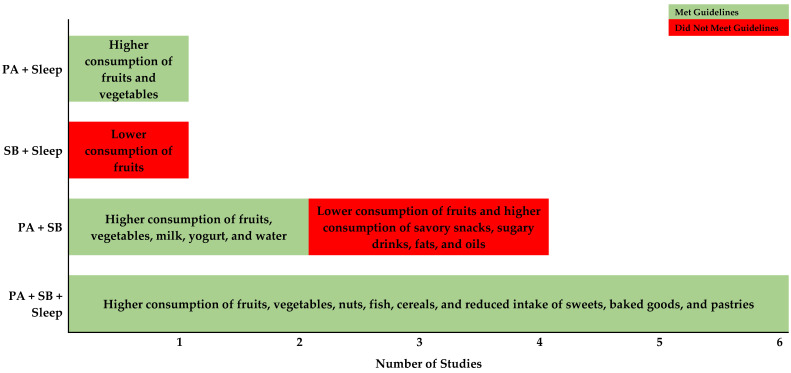
Studies Examine the Relationship Between 24-hour Movement Behaviors and Dietary Outcomes.

In terms of absolute effects, six studies found that meeting physical activity, sedentary time, and sleep recommendations were associated with healthier dietary patterns, including increased intake of fruits, vegetables, nuts, fish, and cereals and reduced consumption of sweets and pastries [28,29,33,34,35,38]. One study observed a dose–response relationship, suggesting that those who met more recommendations had healthier dietary habits [33]. When physical activity and sedentary time were examined together, those adhering to both physical activity and sedentary time guidelines tended to have better dietary habits, such as consuming more fruits and vegetables and fewer sugary drinks and snacks [27,31]. Conversely, increased sedentary time and insufficient sleep were linked to poorer dietary outcomes, particularly reduced fruit consumption [30]. Children who were both active and non-sedentary had better hydration status (consumed more water) compared to those who were sedentary but still active [32]. Additionally, children tended to consume less fruit when sedentary time increased and sleep was inadequate [38]. In Rubín’s (2020) study, they examined the combination of sedentary time and sleep and found that children who met both recommendations tended to consume more fruits and vegetables [31]. We categorized the findings of absolute effects based on combinations of movement behaviors in Figure 2.

## 4. Discussion

Meeting individual movement recommendations is less important for overall health, whereas fulfilling multiple recommendations is linked to better overall health outcomes [1,39,40]. To the best of our knowledge, this is the first systematic review investigating the associations between combinations of 24-hour movement behaviors and dietary outcomes in children and adolescents. Ten cross-sectional studies published between 2017 and 2024 were included. We found that meeting all 24-hour movement behavior guidelines (physical activity, sedentary behavior, and sleep) was consistently linked to higher consumption of fruits, vegetables, nuts, fish, and cereals, while non-adherence was associated with increased intake of sweets and sugary drinks in children and adolescents. The mechanisms behind these associations are likely multifaceted. Specifically, physical activity increases energy expenditure, which not only promotes higher caloric intake but may also encourage the selection of more nutrient-dense foods to replenish energy stores. Furthermore, physical activity can improve appetite regulation, making children more likely to choose healthier foods like fruits and vegetables. Sedentary behavior, particularly screen time, may expose children to extensive advertising for unhealthy foods, leading to passive snacking and increased consumption of sugary and high-fat snacks. The passive nature of these activities may also reduce overall energy expenditure, further promoting unhealthy food choices. Sleep also plays a critical role in regulating hunger and satiety hormones, such as leptin and ghrelin. Insufficient sleep can disrupt these hormones, increasing cravings for high-calorie, high-sugar foods while reducing the body’s ability to feel satiated. Our results also showed that children who met both physical activity and sedentary behavior guidelines were more likely to have better dietary habits, while increased sedentary time and inadequate sleep were associated with poorer outcomes, particularly lower fruit intake. Notably, a dose–response relationship was observed in one study, indicating that meeting more movement behavior guidelines corresponded with progressively better dietary habits. This underscores the importance of adopting a holistic approach when addressing movement behaviors in relation to diet, as it is not just individual behaviors but the combination of these behaviors that appears to have the greatest impact on dietary outcomes. Our findings highlight the need to consider multiple movement behaviors simultaneously when promoting healthy dietary habits in young populations. In this review, we observed some parallel findings with previous review studies that examined the same exposures but different outcomes [19,20,21,26]. Specifically, all the prior studies demonstrated that the most effective combination of movement behaviors is crucial for promoting optimal health in children and adolescents. These benefits include improvements in cardiometabolic health [26], motor skill development, reduced adiposity [19], enhanced mental health indicators [20], and better physical, psychological, and cognitive outcomes [21]. Echoing their findings, our review found that children who adhered to three movement recommendations had the most favorable dietary outcomes compared to their peers who failed to meet these recommendations. It is worth noting that these reviews, including ours, primarily relied on limited evidence from cross-sectional studies, highlighting the need for more comprehensive research to fully understand the lasting impact of these exposures on children’s health outcomes.

Another similarity between the current and previous reviews [19,20,21,26] on children and adolescents is the limited exploration of intermediate combinations of movement behaviors. Most studies in these reviews focused either on children meeting all three recommendations or comparing the most favorable combinations (e.g., high physical activity and low screen time) with those who met none or followed the least favorable combinations (e.g., low physical activity and high screen time). However, less attention has been given to comparing those who met all movement recommendations with those meeting only one or two to identify potential dose–response relationships. Investigating these comparisons could offer important insights into the cumulative benefits of following multiple movement guidelines simultaneously. Additionally, few studies have explored intermediate combinations of movement behaviors, such as high physical activity paired with high screen time, or compared individuals who meet two different combinations (e.g., physical activity and sleep versus physical activity and sedentary time) within the same sample. This highlights a gap in understanding the complexities of children’s movement behaviors. By concentrating primarily on ideal or extreme patterns, we risk overlooking more realistic and common behavior patterns that many children exhibit, which may not align perfectly with ideal movement recommendations but still have meaningful implications for their health. For example, a child engaging in high physical activity but also having high screen time may interact differently with the same health outcomes as one with low screen time, even if both meet two of three overall movement guidelines. This variation in behavior patterns could influence factors such as dietary habits, sleep quality, or overall energy balance [41,42,43]. Without considering these intermediate combinations and their relationships with health outcomes, we fail to capture how nuanced variations in movement behaviors interact to influence overall health. By overlooking these more common, real-world behavior patterns, research may miss critical insights into how different movement behaviors combine to impact dietary outcomes and broader health indicators. Thus, a broader understanding that incorporates the diversity of these behaviors is necessary.

Our review identified notable variations in the measurement of movement behaviors across studies. For younger children, reports were mostly provided by parents or caregivers, often estimating sleep duration and screen time. As children aged, self-reported data became more common for behaviors like screen time and sleep [44,45]. While subjective measures offer valuable insights, they are prone to recall bias and may not accurately reflect actual behaviors [46]. Studies that used objective measures, such as accelerometers, provided more reliable data on physical activity, capturing real-time patterns, intensity, and duration [47]. However, even among these studies [27,30,31,35], differences in wear protocols and data analysis methods introduced variability. These inconsistencies in both subjective and objective measures complicate comparisons across studies, underscoring the need for standardized measurement approaches in future research. Furthermore, most reviewed studies employed generalized linear models to explore associations between 24-hour movement behaviors and dietary outcomes. Since movement behaviors are interrelated, it is essential to account for collinearity between them [48,49]. Analyzing these behaviors in isolation may lead to incomplete conclusions, as they compete for time within a fixed 24-hour period [50,51]. Compositional data analysis, widely used in fields like ecology, nutrition, and movement behavior research, is particularly well-suited for examining time-based data as it allows for analyzing the relative contributions of behaviors, offering a clearer understanding of how physical activity, sedentary time, and sleep, together influence health [49,52,53]. Unfortunately, the studies in this review primarily used regression-based methods and did not employ compositional data analysis, limiting insights into the relative contributions of different movement behaviors to dietary patterns.

Overall, our review makes a significant contribution by synthesizing the relationships between 24-hour movement behaviors and dietary outcomes, showing that children who meet all three movement recommendations have more favorable dietary outcomes. Our review also highlights the importance of addressing multiple movement behaviors simultaneously to promote healthier dietary habits rather than focusing on individual behaviors. This finding reinforces the idea that interventions aiming to improve dietary health in children and adolescents should promote balanced movement behaviors throughout the day. This multifaceted approach may be more effective than single-focus interventions, offering practical insights for parents, caregivers, and policymakers. Nevertheless, we emphasize the need for further research to understand better the causal relationships between movement behaviors and dietary outcomes.

## 5. Strengths and Limitations

The main strengths of this review include its rigorous methodology, prospective registration with PROSPERO, adherence to PRISMA guidelines, expert-designed search strategies, and use of the GRADE framework to assess evidence quality. This review is the first to systematically synthesize the associations between adherence to movement behavior recommendations and dietary outcomes in children and adolescents, addressing a critical gap in the literature. It offers important insights for shaping future interventions and public health guidelines to support optimal growth and health in young populations. However, our review is not without limitations. First, we searched only three databases from 2017 to 2024 and did not include unpublished or non-English sources, which may have led to the exclusion of some relevant studies. Second, although a metanalysis was initially planned, it was not feasible due to the heterogeneity in how movement behaviors and dietary outcomes were measured across the studies. More standardized measurement methods are needed in future studies to allow for more robust comparisons and the synthesis of results. Lastly, our findings are based on “very low” quality evidence, primarily due to the cross-sectional design of all the included studies, which restricts the ability to draw definitive conclusions. While the cross-sectional design limits the ability to infer causality, the observed associations provide valuable insights into the prevalence of healthier dietary patterns among those meeting 24-hour movement behavior guidelines. These findings are hypothesis–generating and underscore the need for future longitudinal and experimental research to explore these relationships further. Future research using experimental designs is strongly encouraged to confirm causality.

## 6. Conclusions

Our review observed that children and adolescents, compared to their peers, who adhered to all three 24-hour movement guidelines had higher consumption of fruits, vegetables, nuts, fish, and cereals and reduced intake of sweets and baked goods. Meeting both physical activity and sedentary behavior guidelines was associated with higher odds of consuming fruits and vegetables while failing to meet these guidelines was linked to greater consumption of unhealthy snacks and sugary beverages. Additionally, meeting both physical activity and sleep guidelines was positively associated with better fruit and vegetable intake, while increased sedentary behavior and inadequate sleep were negatively correlated with fruit consumption. These findings underscore the importance of promoting balanced movement behaviors to support healthier eating patterns in young populations. However, the quality of the current evidence is constrained by limitations in study design and the predominance of cross-sectional data, which makes it difficult to establish clear causal relationships. To better understand the dietary benefits of various movement behavior combinations, high-quality longitudinal and intervention studies focusing on intermediate movement behavior combinations are urgently needed. Furthermore, using advanced statistical methodologies could provide deeper insights into how movement behaviors interact to influence dietary outcomes. Future research will be essential for guiding the development of comprehensive guidelines that optimize movement patterns to improve dietary health in children and adolescents.

## Figures and Tables

**Table 1:** Characteristics of included studies (n = 10).

Reference (Author, Year, Location)	Study Design	No. of Participants and Age Range (yrs)	Statistical Analysis	Physical Activity Measure	Sedentary Measure	Sleep Measure	Dietary Outcome and Measure	Findings on the Combinations of 24-Hour Movement Behaviors and Their Associations Dietary Outcomes
Caetano et al. (2022), Brazil [27]	Cross-sectional study	309 adolescents, 14–16 yrs	Latent class analysis	Accelerometer (GT3X)	Accelerometer-measured sedentary time; Screen time questionnaire	N/A	Weekly frequency of fruits, vegetables, and sugars intake via the Risk Behaviors of Adolescents from Santa Catarina—COMPAC questionnaire	Inactive and sedentary behaviors were associated with low fruit intake.
López-Gil et al. (2022), Spain [28]	Cross-sectional study	3772 minors, 0–14 yrs	Logistic regression	Parent-reported physical activity via a modified short version of the International Physical Activity Questionnaire	Parent-reported recreational screen time	Parent-reported sleep duration	Frequency of food group consumption (e.g., fruits, vegetables, dairy) via the Spanish Healthy Eating Index (S-HEI)	Meeting all 24-hour movement guidelines was associated with a higher quality diet. Children in the highest tertile of the S-HEI were more likely to meet these guidelines. Additionally, children who achieved adequate sleep and had lower screen time showed better adherence to a healthy diet.
López-Gil et al. (2024), Spain [29]	Cross-sectional study within the SENDO project cohort	822 preschool children, 4–5 yrs	Generalized linear models	Parent-reported physical activity	Parent-reported screen time, including television and computer use	Parent-reported sleep duration	Food consumption via the KIDMED index (Mediterranean Diet Quality Index)	Children who met all three 24-hour movement recommendations were more likely to consume fruits, vegetables, and nuts regularly compared to children who did not meet the recommendations.
Moradell et al. (2023), Spain [30]	Cross-sectional study	1448 children and adolescents, 12–18 yrs	Generalized linear models	Accelerometer (GT3X)	Screen time questionnaire	N/A	Food consumption categories (Fruits, Vegetables, Dairy, Snacks, etc.) via the HELENA-DIAT 24-hour dietary recall (24-HDR)	In both males and females, the intake of savory snacks was higher among individuals who did not meet any of the physical activity and sedentary time recommendations. Males who met both recommendations were more likely to consume milk, yogurt, and water, while those not meeting recommendations were more likely to consume sugar-sweetened beverages. For females, failing to meet the recommendations was associated with lower fruit and vegetable intake and a higher intake of fats and oils.
Rubín et al. (2020), Czech Republic [31]	Cross-sectional study	355 children aged 8–13 yrs and 324 adolescents aged 14–18 yrs	Multi-level multivariable logistic regression	Accelerometer (wGT3X-BT and GT9X Link)	Self-reported recreational screen time	Accelerometer-based sleep duration	Self-reported fruit and vegetable intake	Children meeting combinations of two movement behaviors (i.e., physical activity and sleep, physical activity and screen time, or screen time and sleep) were associated with higher fruit and vegetable intake.
Perales-García et al. (2018), Spain [32]	Cross-sectional study	242 children, 7–12 yrs	Logistic regression	Parent-reported physical activity	Parent-reported screen time	N/A	Dietary water intake and hydration status via a 3-day dietary record and 24-hour urine osmolality	Children who were both non-sedentary and active had better hydration status compared to sedentary active children.
Sampasa–Kanyinga et al. (2022), Canada [33]	Cross-sectional study	12,759 children and adolescents, 11–20 yrs	Multivariable ordered logistic regression	Self-reported physical activity	Self-reported screen time, (video games, TV, movies, texting, internet)	Self-reported sleep duration	Frequency of breakfast consumption and fruit and vegetable intake, via self-reported questionnaires	Compliance with all three movement behavior recommendations was associated with more frequent breakfast consumption and higher fruit and vegetable intake compared to those who met none of the recommendations. A dose–response relationship was observed, where meeting more recommendations was linked to increased frequency of breakfast and fruit and vegetable consumption.
Tambalis et al. (2024), Greece [16]	Cross-sectional study	177,091 children, 8–17 yrs	Binary logistic regression	Self-reported questionnaire	Screen time and sedentary behavior questionnaire	Self-reported sleep duration	Measured using the KIDMED index (Mediterranean Diet Quality Index) with a focus on fruit consumption. Other dietary components include fish, vegetables, legumes, and dairy consumption.	Increased fruit consumption was positively correlated with reduced sedentary time, more sleep, and higher levels of physical activity. Sedentary time and inadequate sleep were negatively associated with fruit consumption.
Tapia-Serrano et al. (2022), Spain [34]	Cross-sectional study	1391 children and adolescents, 11–16 yrs	Binary logistic regression	Self-reported physical activity via the Physical Activity Questionnaire for Adolescents (PAQ-A)	Self-reported recreational screen time via the Youth Leisure Sedentary Behavior Questionnaire (YLSBQ)	Self-reported sleep duration	Food consumption via the KIDMED index (Mediterranean Diet Quality Index)	Adolescents who met all three 24-Hour Movement Guidelines showed greater adherence to the Mediterranean diet compared to those who did not. They consumed more fruits, vegetables, fish, and cereals, while reducing their intake of baked goods, pastries, sweets, and candies.
Thivel et al. (2018), Canada [35]	Cross-sectional study	5873 children, 9–11 yrs	Multilevel linear mixed model	Accelerometer (GT3X)	Self-report screen time	Accelerometer-based sleep duration	Dietary patterns via the Food Frequency Questionnaire (FFQ)	Meeting all three movement behavior recommendations was associated with the healthiest dietary patterns.

**Table 2:** Quality Assessment and Absolute Effects of Movement Behaviors on Dietary Behaviors.

No. of Study (Participants)	Design	Quality Assessment					Quality	Absolute Effect
	Risk of Bias		Inconsistency	Indirectness	Imprecision	Other		
10 (204,386)	Cross-sectional	Serious risk of bias	No serious inconsistency	No serious indirectness	No serious indirectness	Dose-response	VERY LOW	**MEET PA + SB + SLEEP RECOMMENDATIONS** Six studies found that meeting all 24-hour movement guidelines (compared with meeting none) was consistently associated with healthier dietary outcomes, including a higher quality diet [28], increased consumption of fruits [38], vegetables, nuts [29,33], fish, cereals, and a reduction in sweets, baked goods, pastries [34], and a healthiest overall dietary patterns [35]. **MEET PA + SB RECOMMENDATIONS** Four studies explored the relationship between the combination of PA and SB and dietary outcomes. One study found that inactive and sedentary behaviors were linked to low fruit intake [27]. Conversely, another study found that children who met the PA and SB guidelines had higher odds of consuming fruits and vegetables [31]. Another one observed both males and females who did not meet PA and SB recommendations consumed more savory snacks. Males meeting both recommendations favored milk, yogurt, and water, while others consumed more sugar-sweetened beverages. Females not meeting recommendations had lower fruit and vegetable intake and higher fats and oils consumption [30]. Interestingly, one study found that children who were both active and non-sedentary had better hydration status (consumed more water) compared to those who were sedentary but still active [32]. **MEET SB + SLEEP RECOMMENDATIONS** One study also examined the combination of SB and sleep in relation to dietary patterns, and found that increased sedentary time and inadequate sleep were negatively associated with fruit consumption [38]. **MEET PA + SLEEP RECOMMENDATIONS** An above study also found that children who met both physical activity and sleep recommendations tended to consume more fruits and vegetables [31].

Note. PA = physical activity, SB = sedentary behavior (or screen time or sedentary time).

## Supplementary Materials

The following supporting information can be downloaded at: https://www.mdpi.com/article/10.3390/nu16213678/s1, A full description of the strategy used for each database.
