# Supplementary material for: Associations Between Combinations of 24-Hour Movement Behaviors and Dietary Outcomes in Children and Adolescents: A Systematic Review
Source: Nutrients. 2024 Oct 29;16(21):3678. doi: 10.3390/nu16213678 (PMC11547975; doi:10.3390/nu16213678)
Supplement: Supplementary file 1 [file nutrients-16-03678-s001.zip › nutrients-3249464-supplementary.pdf]

Supplementary Materials: Search Strategies

Database (MEDLINE, Embase, PsycINFO)
Note: Searches were conducted using an Ovid multi-database search, with duplicate records removed online, prioritizing MEDLINE first, followed by Embase.

MEDLINE
1. Physical Activity.mp.
2. exp Exercise/
3. exp Exercise Movement Techniques/
4. exp Exercise Therapy/
5. Physical Exertion/
6. exp "Physical Education and Training"/
7. exp Sports/
8. (sport$ or bicycl$ or swim$ or walk$ or run$ or jog$).tw,kf.
9. (physical$ adj2 activ$).tw,kf.
10. (aerobic adj2 (train$ or active$)).tw,kf.
11. "Play and Playthings"/ and (activ* or outdoor*).tw,kf.
12. ((activ* or outdoor*) adj3 play*).tw,kf.
13. playground*.tw,kf.
14. active.ti. and (space* or behavio?r* or transport* or commut* or neighbo?rhood* or park* or game* or gaming or lifestyle).mp.
15. (active adj3 (space* or behavio?r* or transport* or commut* or neighbo?rhood* or park* or game* or gaming or lifestyle)).tw,kf.
16. or/1-15
17. Sleep.mp.
18. exp *Sleep Apnea Syndromes/ and (apnea or apnoea).ti.
19. 17 not 18
20. Sedentary Lifestyle/
21. bed rest.mp. or (sedentary or inactiv* or sitting or (lack adj2 activity) or (low adj3 energy expend*)).ti,ab,kf.
22. ((chair or stroller or car or automobile* or auto or bus or motor vehicle* or indoor* or in-door* or screen or computer) adj time).ti,ab,kf.
23. Television/ or Computers/ or Video games/ or Software/ or Videodisc Recording/ or Cartoons as Topic/ or Motion Pictures as Topic/ or exp Internet/ or exp Computers, handheld/ or Communications Media/ or Mass Media/
24. (television or tv or screentime or ((watch* or view*) adj2 (dvd* or video*)) or screen media or social media or video gam* or videogam* or computer gam* or electronic gam*).ti,ab,kf.
25. (screen based entertainment or screen-based entertainment or smartphone* or ipad or apps or app or mobile applications).ti,ab,kf.
26. or/20-25
27. 16 and 19 and 26
28. 27 and (child* or adolesc*).mp.
29. limit 28 to (yr="2017 - 2024" and (english) and (journal article or published erratum or "retraction of publication"))

30. exp physical activity/ or exp exercise/ or exp kinesiotherapy/ or physical education/ or exp sport/

Embase
31. (sport* or bicycl* or swim* or walk* or run* or jog*).tw,kw.
32. (aerobic adj2 (train$ or active$)).tw,kw.
33. Play/ and (activ* or outdoor*).tw,kw.
34. (((activ* or outdoor*) adj3 play*) or playground*).tw,kw.
35. active.ti. and (space* or behavio?r* or transport* or commut* or neighbo?rhood* or park* or game* or gaming or lifestyle).mp.
36. (active adj3 (space* or behavio?r* or transport* or commut* or neighbo?rhood* or park* or game* or gaming or lifestyle)).tw,kw.
37. or/31-36
38. Sleep/
39. Sleep Time/
40. (sleep adj3 duration).tw.
41. exp Sleep Disordered Breathing/ and (apnea or apnoea).ti.
42. (or/38-40) not 41
43. sedentary lifestyle/
44. bed rest.mp. or (sedentary or inactiv* or sitting or (lack adj2 activity) or (low adj3 energy expend*)).ti,ab,kw.
45. ((chair or stroller or car or automobile* or auto or bus or motor vehicle* or indoor* or in-door* or screen or computer) adj time).ti,ab,kw.
46. video game/ or software/ or videorecording/ or movie/ or exp mass communication/ or television viewing/ or television/ or exp computer/ or internet addiction/ or mobile application/ or exp mobile phone/
47. (television or tv or screentime or ((watch* or view*) adj2 (dvd* or video*)) or screen media or social media or video gam* or videogam* or computer gam* or electronic gam*).ti,ab,kw.
48. (screen based entertainment or screen-based entertainment or smartphone* or ipad or apps or app or mobile applications).ti,ab,kw.
49. or/43-48
50. 37 and 42 and 49
51. 50 and (child* or pediatr* or paediatr* or teen* or adolescen*).mp.
52. limit 51 to (embase and (english) and yr="2017 -Current")
53. physical activity/ or exp exercise/ or activity level/ or movement therapy/ or dance therapy/ or mind body therapy/ or energy expenditure/ or physical education/ or exp sports/

PsycINFO
54. (sport* OR bicycl* OR swim* OR walk* OR run* OR jog*).tw,id.
55. (physical* adj2 activ*).tw,id.
56. (aerobic adj2 (train* OR active*)).tw,id.
57. (childhood play behavior/ OR childhood play development/ OR games/ OR recreation/) AND (activ* OR outdoor*).tw,id.
58. ((activ* OR outdoor*) adj3 play*).tw,id.
59. playgrounds/ OR playground*.tw,id.

60. active.ti. AND (space* OR behavio?r* OR transport* OR commut* OR neighbo?rhood* OR park* OR game* OR gaming OR lifestyle).tw,id.
61. (active adj3 (space* OR behavio?r* OR transport* OR commut* OR neighbo?rhood* OR park* OR game* OR gaming OR lifestyle)).tw,id.
62. OR/54-61
63. Sleep/ OR Sleep Deprivation/
64. (sleep adj3 duration).tw.
65. *Sleep Apnea/ AND (apnea OR apnoea).ti.
66. (63 OR 64) NOT 65
67. Sedentary Behavior/
68. bed rest.mp. OR (sedentary OR inactiv* OR sitting OR (lack adj2 activity) OR (low adj3 energy expend*)).tw.
69. ((chair OR stroller OR car OR automobile* OR auto OR bus OR motor vehicle* OR indoor* OR in-door* OR screen OR computer) adj time).tw.
70. Screen Time/ OR Television Viewing/ OR exp Computers/ OR exp Computer usage/ OR Computer Games/ OR Digital Gaming/ OR Internet/ OR exp Communications Media/ OR Internet Addiction/ OR exp Internet Usage/ OR exp Mobile Devices/ OR Smartphone/ OR Mobile Applications/
71. (television OR tv OR screentime OR ((watch* OR view*) adj2 (dvd* OR video*)) OR screen media OR social media OR video gam* OR videogam* OR computer gam* OR electronic gam*).tw.
72. (screen based entertainment OR screen-based entertainment OR smartphone* OR ipad OR apps OR app OR mobile applications).tw.
73. OR/67-72
74. 62 AND 66 AND 73
75. 74 AND (child* OR adolescen* OR teen* OR youth* OR "early childhood" OR preschool*).mp.
76. limit 74 to (100 childhood <birth to 12 yrs> OR 120 school age <6 to 12 yrs> OR 140 adolescence <13 to 18 yrs>)
77. 75 OR 76
78. limit 77 to (english)
79. limit 78 to ("erratum/correction" OR journal article)
80. 79 OR (78 AND retraction.ti.)
81. limit 80 to yr="2017 -Current"

The following lines are part of an advanced search strategy on a database platform like Ovid, where search results from three databases are merged
82. 29 use medall
83. 52 use emczd
84. 81 use psyh
85. or/82-84
